# A Two-Stage Hybrid Default Discriminant Model Based on Deep Forest

**DOI:** 10.3390/e23050582

**Published:** 2021-05-08

**Authors:** Gang Li, Hong-Dong Ma, Rong-Yue Liu, Meng-Di Shen, Ke-Xin Zhang

**Affiliations:** 1 School of Business Administration, Northeastern University, Shenyang 110819, China; 1901921@stu.neu.edu.cn (H.-D.M.); 1901920@stu.neu.edu.cn (R.-Y.L.); 1801915@stu.neu.edu.cn (M.-D.S.); kexinzkx@126.com (K.-X.Z.); 2 School of Economics, Northeastern University at Qinhuangdao, Qinhuangdao 066004, China; 3 Institutes of Science and Development, Chinese Academy of Sciences, Beijing 100190, China

**Keywords:** default discrimination, feature selection, deep forest, credit score, credit loan

## Abstract

**Background:** the credit scoring model is an effective tool for banks and other financial institutions to distinguish potential default borrowers. The credit scoring model represented by machine learning methods such as deep learning performs well in terms of the accuracy of default discrimination, but the model itself also has many shortcomings such as many hyperparameters and large dependence on big data. There is still a lot of room to improve its interpretability and robustness. **Methods:** the deep forest or multi-Grained Cascade Forest (gcForest) is a decision tree depth model based on the random forest algorithm. Using multidimensional scanning and cascading processing, gcForest can effectively identify and process high-dimensional feature information. At the same time, gcForest has fewer hyperparameters and has strong robustness. So, this paper constructs a two-stage hybrid default discrimination model based on multiple feature selection methods and gcForest algorithm, and at the same time, it optimizes the parameters for the lowest type II error as the first principle, and the highest AUC and accuracy as the second and third principles. GcForest can not only reflect the advantages of traditional statistical models in terms of interpretability and robustness but also take into account the advantages of deep learning models in terms of accuracy. **Results:** the validity of the hybrid default discrimination model is verified by three real open credit data sets of Australian, Japanese, and German in the UCI database. **Conclusions:** the performance of the gcForest is better than the current popular single classifiers such as ANN, and the common ensemble classifiers such as LightGBM, and CNNs in type II error, AUC, and accuracy. Besides, in comparison with other similar research results, the robustness and effectiveness of this model are further verified.

## 1. Introduction

In recent years, research on the default discriminant model has received extensive attention from researchers and financial institutions. The accuracy of its discriminant greatly affects the risk control and profitability of financial institutions. To prevent the losses caused by bad credit decisions, many recent studies are devoted to finding ways to improve the accuracy of the default discrimination model. Most of these studies focus on optimizing the model by adjusting the model parameters to improve the prediction accuracy.

At present, the research on the default discrimination model is mainly based on two aspects. On the one hand, there are traditional default discrimination models, such as Z-score Model [1], Probit analysis method [2], and Logistic analysis model [3]. This kind of model has great advantages in variable interpretability and robustness. It is one of the widely used models in the field of personal credit evaluation. Its disadvantage is that it cannot handle high-dimensional data. On the other hand, there are artificial intelligence models, such as artificial neural network (ANN) [4], support vector machine (SVM) [5], decision tree (DT) [6], and so on. Its advantages are high prediction accuracy and no strict requirements on the distribution of data. Its disadvantage is that the robustness of the model is poor. At the same time, because the model is a black box operation, the interpretability of the results of default discrimination is not good.

Various types of default discrimination model have their advantages and disadvantages. At the same time, with more and more data acquisition channels, the dimensionality of variables is getting higher and higher. Multicollinearity between variables will reduce the explanatory and predictive accuracy of the model. So, it is very important to select a set of feature subsets with more predictive information. Reducing the number of irrelevant or redundant features greatly reduces the training and running time of the classification model and can promote data visualization and data understanding. Besides, it can reduce acquisition and storage requirements, and break the curse of dimensionality, and improve prediction performance.

To solve the shortcomings of the single model, research of the default discrimination model gradually turns to the ensemble model. The ensemble model can not only absorb the advantages of the basic model but also reduce the shortcomings of the basic model. It has become the current research hotspot of the personal credit default discrimination model. The two most common forms of ensemble models are hybrid methods and ensemble methods (hybrid classifiers and classifier ensemble). The hybrid method refers to the combination of feature selection or parameter optimization before classification, and the ensemble method refers to the ensemble of multiple classifiers [7]. Many existing ensemble models are changes or improvements of the two methods [8,9]. As part of data preprocessing, feature selection algorithms have been proven by many researchers to improve the performance of machine learning models [10].

However, although a lot of studies have been devoted to hybrid models and ensemble models, a few studies have examined the interpretability of feature selection methods and the influence of several feature selection methods on the problem of default discrimination. Besides, existing research on deep learning models is mostly based on neural networks. Although deep learning models have been proven to perform well in many fields, they still have many shortcomings: many hyperparameters, training requires a lot of training data, and determination the structure of the neural network before training.

To make up for the shortcomings of the above research and improve the interpretability, classification performance, and robustness of the credit scoring model, this paper establishes a new two-stage hybrid model combining multiple feature selection methods and gcForest. This model considers the differences and complementarities between traditional statistical models and artificial intelligence models and combines the two to complement each other.

In the hybrid model, five interpretable feature selection algorithms are selected in three types of feature selection methods: filtering, packaging, and embedding: (1) Full-variable Logistic regression; (2) Stepwise regression based on AIC criterion; (3) Stepwise regression based on BIC criterion; (4) Lasso-logistic regression; (5) Elastic Net Logistic regression. For each feature selection algorithm, its performance is tested according to type II error, AUC and accuracy. The first principle is the lowest type II error, and the highest AUC and accuracy are second and third principles. Then, using the feature set obtained in the feature selection process, we combine different algorithms to construct different default discrimination models.

To build a default discrimination model with better discriminative performance and robustness, we introduce gcForest [11], which has an excellent performance in many fields, into the credit field. Existing studies have shown that tree-based ensemble machine learning techniques such as random forest (RF) [12] have advantages in dealing with nonlinear classification problems and overfitting. Zhou et al. (2017) proposed a new tree-based ensemble method, gcForest, and proved that it has highly competitive performance with deep neural networks (DNNs) in a wide range of tasks. Although DNNs is powerful, it also has many shortcomings [11]. Firstly, DNNs has too many hyperparameters, and its learning performance depends heavily on parameter adjustments. Secondly, network architecture must be determined before training, and it is more complicated to adjust the structure. Random forest [13] or XGBoost [14] have won many Kaggle competition tasks. On this basis, Zhou et al. have deeply analyzed the key to success of the deep model and believed that there are three key features behind the excellent performance of DNNs, namely, layer-by-layer processing, feature conversion within the model, and sufficient model complexity [15]. Zhou et al. have tried to give these features to the deep model of the non-neural network, and then designed a gcForest based on decision tree. Compared with deep learning, SVM, Logistic regression in image classification, face recognition, music classification and other fields, Zhou et al. have proved the effectiveness of gcForest. GcForest has also been successfully widely studied in many fields such as medicine and social science [16,17].

Although gcForest has been proven to perform well in many fields, it has not yet been applied in the field of personal credit default discrimination. GcForest uses a cascade structure to process features layer by layer to further improve the learning ability of the model and obtain better classification performance. To reduce the risk of overfitting, the class vector generated by each forest in the cascade structure is generated by k-fold cross-validation. Compared with most DNNs models with fixed complexity, gcForest adaptively determines its model complexity by terminating training at an appropriate time. This makes it applicable to training data of different scales, and is not limited to large-scale training data. So, this article applies gcForest to the identification of personal credit default and builds a personal credit default discrimination model with better predictive performance and robustness.

The rest of this article is organized as follows. Section 2 reviews the relevant methods used in this article. Section 3 describes the construction process of the proposed hybrid model based on gcForest. Section 4 gives the experimental setup in detail, including data set description, preprocessing, and performance evaluation. Section 5 is the empirical research process of the two-stage hybrid model and the analysis of the experimental results. Section 6 summarizes this article.

## 2. Literature Review

This part mainly introduces the application of feature selection and deep learning model in credit scoring.

### 2.1. Feature Selection

Generally speaking, the data set contains multiple different features, which may include irrelevant or redundant features, making it difficult to train the model, and reducing model interpretability and accuracy. Therefore, feature selection has become a basic task in default discrimination. Feature selection methods can be divided into three categories: filtering method, packaging method, and embedding method [18]. These three methods have their own advantages and disadvantages. Researchers try to improve the performance of the classifier by combining them [19]. Chen et al. (2010) compared four feature selection methods such as LDA and rough set, and the experimental results showed that the prediction result with feature selection process is better than the prediction result without feature selection process. It also proved the effectiveness of feature selection to improve the performance of the classifier [20]. Koutanaei et al. (2015) used four feature selection methods such as principal component analysis (PCA) combined with ensemble learning classification algorithms to study hybrid data mining models and proved that the use of feature selection algorithms and ensemble classifiers can improve the model’s performance in the default discrimination problem [21]. Liang et al. (2015) pointed out that most studies only focus on the application of specific feature selection methods in bankruptcy prediction or default discrimination problems. Therefore, they studied the impact of feature selection of three filtering methods and two packaging methods on financial distress prediction. The experimental results showed that there was no optimal combination of feature selection methods and classifiers on the four data sets [22]. Dahiya et al. (2017) used two feature selection methods, chi-square test and PCA, to sort and select important features in the data set and proved that the hybrid model based on feature selection and machine learning technology significantly improved the accuracy of the independent model [23]. Trivedi (2020) compared and analyzed different feature selection techniques and different machine learning classifiers by using four feature selection techniques such as information gain and five machine learning classifiers such as Bayes on German data set, determined the best combination of feature selection technology and machine learning classifier on this data set [24].

### 2.2. Application of Deep Learning Model in Credit Scoring

Existing studies have shown that compared with traditional statistical models and classic machine learning models, the application of deep learning technology in financial forecasting has been significantly improved, but in terms of credit scoring, deep learning technology has not been widely used [25]. Wang et al. (2018) based on online operation behavior data of borrowers in P2P lending proposed a consumer credit scoring method based on the LSTM model and evaluated the method on a real data set [26]. Kim et al. (2019) proposed a convolutional neural networks (CNNs) architecture for classifying the loan status of borrowers in P2P lending to automatically select complex features and improve model performance [27]. Pawiak et al. (2019) proposed a support vector machine deep genetic cascade ensemble classifier (DGCEC) based on evolutionary computation, ensemble learning, and deep learning technology, which could effectively classify borrowers, accept, or reject applications. In the empirical study, Australian Statlog data set was used to verify the performance of the model [28]. Zhang et al. (2020) aimed at the problem that P2P credit data usually contains dense numerical features and sparse category features, proposed an online integrated credit scoring model (OICSM) that combines a GBDT and a neural network. The scoring model could deal with the two types of features more effectively, and the effectiveness and superiority of the model were verified through empirical research [29]. Plawiak et al. (2020) proposed a Deep Genetic Hierarchical Network of Learners (DGHNL) credit scoring model integrating SVM, KNN, probabilistic neural network, and fuzzy system, and the validity of the method was proved by German credit data set in UCI database [30]. To deal with the imbalance of credit data, Shen et al. (2021) developed a new deep learning ensemble credit risk assessment model that combined the LSTM algorithm and the AdaBoost algorithm, and compared the performance of the proposed model and other widely used credit scoring models on two imbalanced credit data sets [31].

Generally speaking, the number of features in the credit scoring system of commercial banks and other financial institutions cannot be too many. Although typical machine learning algorithms have high predictive performance, most algorithms lack interpretability. To solve this problem, more and more researchers have studied feature selection in recent years. Previous studies have shown that a single feature selection method cannot handle all classifiers and data sets well [32]. Although the existing studies have begun to focus on combining multiple feature selection methods to improve the performance of the classifier, there is a lack of interpretable optimal feature set determination method analysis. Besides, existing studies have found that in the field of default discrimination, deep learning can reveal the complex relationship between credit data variables, making its performance better than traditional statistical methods and machine learning methods [31]. However, existing research on deep learning models still has shortcomings such as many hyperparameters, requiring a large amount of training data, and determination the structure of the neural network before training. Therefore, to make up for the deficiencies of the existing research, gcForest with excellent performance in multiple fields is introduced into the field of default discrimination and combined with a variety of feature selection methods to construct a two-stage hybrid model with better interpretability, robustness, and classification performance in this article.

## 3. Construction of Hybrid Default Discriminant Model Based on GcForest

**Figure 1:**
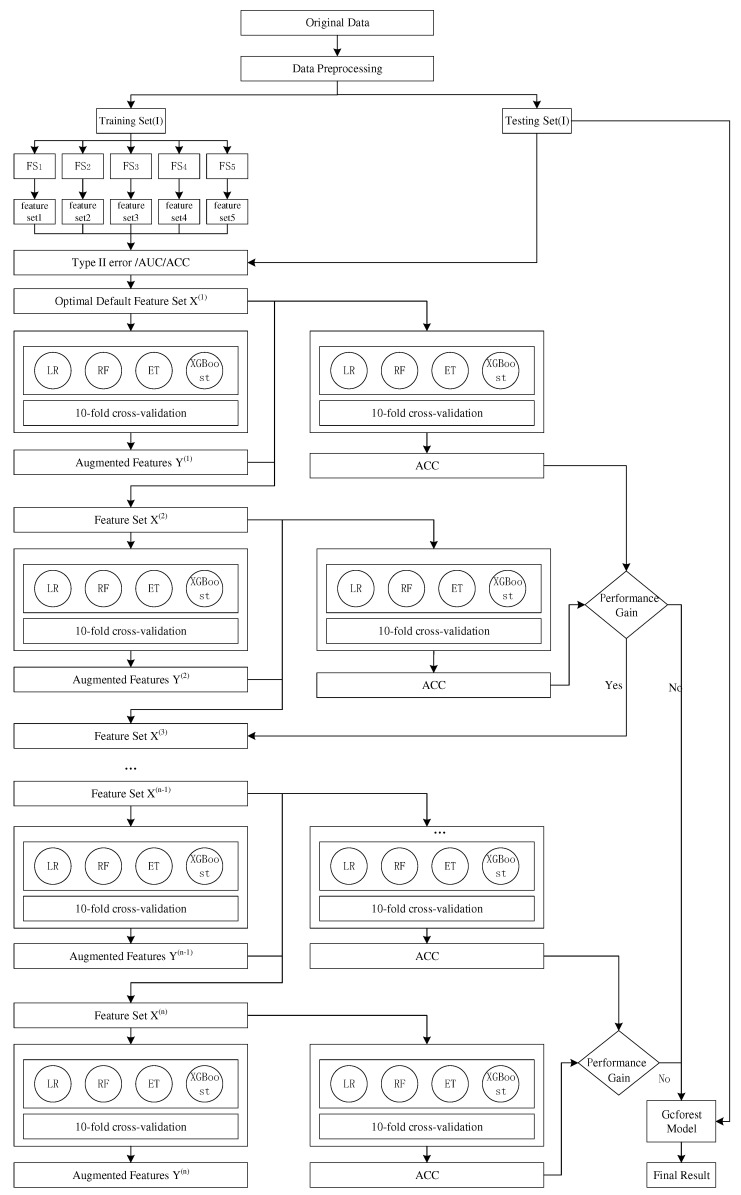
Model frame diagram.

To construct a default discrimination model with better interpretability, accuracy, and robustness, this section proposes a two-stage hybrid model that combines multiple feature selection methods and the default discrimination model based on gcForest. Figure 1 shows the framework of the proposed model. It is mainly divided into two stages. The first stage is determination of the optimal feature set; the second stage is construction of the default discrimination model based on gcForest. This research divides the original training data set into a training set (I) and a test set (I) in the first stage. In the second stage of constructing the default discrimination model, we use the 10-fold cross-validation method to evaluate classification performance of the model.

The first stage: determination of the best feature set. Firstly, the best feature set should have good interpretability. Secondly, the lowest type II error is the first principle and the highest AUC and accuracy are the second and third principles, respectively. The detailed steps are as follows:

(1) Data preprocessing. Data preprocessing is very important for the efficiency and accuracy of the classification model. In empirical work, we use multistage data preprocessing technology and use the processed data set in the feature selection process.
(2) Feature selection. No one feature selection algorithm can be applied to all data sets. Instead of using a single feature selection algorithm, we have selected five different feature selection methods among three types of feature selection methods: filtering, packaging, and embedding. Then, we find a feature selection algorithm suitable for the data set and a set of optimal feature subsets, so that the classification algorithm can obtain better performance in the second stage (the modeling stage). The feature selection methods used at this stage are as follows: (1) Full-variable Logistic regression; (2) Stepwise regression based on AIC criterion; (3) Stepwise regression based on BIC criterion; (4) Lasso-logistic regression; (5) Elastic Net Logistic regression. The Akaike information criterion (AIC criterion) was founded and developed by Japanese statistician Akaike Hiroji in 1974. It is based on the concept of entropy, which can weigh the complexity of the estimated model and the goodness of the model to fit the data. The AIC criteria is shown in Equation (1):
  (1) $$AIC=-2\log L(\hat{\theta })+2p,$$
  where the first term on the right side of the Equation (1) is the negative log-likelihood loss, $\hat{\theta }$ is the likelihood function of θ, and the second term is the penalty for the number *p* of model parameters (model complexity). The smaller the value of AIC, the better. Bayesian Information Criterion (BIC criterion), like AIC criterion, is used to maximize the fitting of the likelihood function and is shown in Equation (2):
  (2) $$BIC=-2\log L(\hat{\theta })+p\log n,$$
  where *n* is the number of samples. Other variables have the same meaning as Equation (1). The smaller the value of BIC, the better. Among them, the Full-variable Logistic regression is the filtering method, and Stepwise regression based on AIC criterion and Stepwise regression based on BIC criterion are the packaging methods, and Lasso-logistic regression and Elastic Net Logistic regression are the embedding methods.
(3) Feature set evaluation and optimal feature set determination. At this stage, all feature selection methods are studied, and the constructed five groups of default discriminant feature sets are used for Logistic regression, and the feature selection methods are analyzed through Logistic regression classification type II error, AUC, and accuracy. In the evaluation, the first principle is the lowest type II error, and the second and third principles are the highest AUC and accuracy respectively to select a set of optimal default discrimination features for the second stage of model.

The second stage: using gcForest combined with the optimal feature subset of the first stage to construct a default discrimination model.

Multi-Grained Cascade Forest (gcForest) is a deep model based on decision tree, which uses a cascade structure to let gcForest do representation learning. When input data has high-dimensional features, its characterization learning ability can be further improved through multigranularity scanning. GcForest can adaptively determine the number of cascading layers according to the data set and determine the complexity of the model by itself. Besides, gcForest has fewer hyperparameters than DNNs and has relatively good robustness for hyperparameter settings. In most cases, even if it encounters different data in different fields, it can still use the acquiescent setting to achieve excellent results.

Representation learning in DNNs mainly relies on layer-by-layer processing of original features. Deep learning imitates the mechanism of the human brain to interpret data and combines low-level features to form more abstract high-level features, making it more and more able to express internal laws. Inspired by this, gcForest adopts a cascade structure, in which each layer in the cascade receives characteristic information processed by the previous stage and outputs the processing result of this stage to the next stage.

Each level of the original cascade includes two random forests and two Extra-Trees. In our credit default discrimination problem, there are two categories of borrowers: default and nondefault. Since there are two categories to predict the final state of the borrower, each forest will output a two-dimensional category vector, which is then connected with the original input vector as the input of the next layer, and so on. Each forest in the last layer will output a two-dimensional category vector, and then we average the two-dimensional category vectors, and finally get a two-dimensional category vector.

An example of the calculation process is as follows:

We give a sample of the *t*-th borrower $O_{t}$ = ($X_{t}$,$Y_{t}$), $X_{t}$ = ($X_{t1}$, $X_{t2}$,…, $X_{tl}$, …, $X_{tn}$) represents the *n* feature set of the *t*-th borrower, where $X_{tl}$ represents the *l*-th characteristic value of the *t*-th borrower. $Y_{t}$ = (0,1) represents the default status of the *t*-th borrower, which is a binary variable. When $Y_{t}$ = 0, it means that the borrower is in nondefault status, and when $Y_{t}$ = 1, it means that the borrower is in default status.

We suppose the initial feature set of the *t*-th borrower $X_{t}$ = ($X_{t1}$, $X_{t2}$,…, $X_{tl}$, …, $X_{tn}$) as the first layer of gcForest cascade structure $X_{t}^{\left(1\right)}=\left(X_{t1},X_{t1},\ldots X_{tn}\right)$, *t* = 1, 2, …, *w*. The value of *w* in the cascade structure is automatically determined. When a new layer is extended, the performance of the entire cascade will be estimated on the verification set. If there is no significant performance improvement, the training process will be terminated. We suppose there are *z* forests in each layer and each forest contains *m* decision trees. For each decision tree in the random forest, the leaf node corresponding to the sample $O_{i}$ can be obtained, and the proportion of all training samples in the leaf node in different categories is taken to obtain a two-dimensional vector, which represents the proportion of the two categories. Assuming that the decision tree belongs to the *k*-th tree in the *j*-th forest of the *i*-th layer, the two-dimensional class vector obtained from the decision tree can be expressed as $Y_{t}^{ijk}=\left(p_{t}^{ijk},q_{t}^{ijk}\right)$ and satisfy $p_{t}^{ijk}+q_{t}^{ijk}=1$. Then, we average the two-dimensional vectors obtained from all trees in the forest to generate an estimate of the distribution of the class. Then, the calculation process of the final class vector $Y_{t}^{ij}$ obtained from the *j*-th forest of the *i*-th layer is shown in Equation (3):

(3) $$Y_{t}^{ij}=\frac{1}{m}\sum_{k=1}^{m}Y_{t}^{ijk}=\left(p_{t}^{ij},q_{t}^{ij}\right),$$

where,

(4) $$p_{t}^{ij}=\frac{1}{m}\sum_{k=1}^{m}p_{t}^{ijk},$$

(5) $$q_{t}^{ij}=\frac{1}{m}\sum_{k=1}^{m}q_{t}^{ijk}.$$

We connect the class vectors $Y_{t}^{ij}$ generated by different random forests in the same layer as the enhancement feature of the *t*-th borrower. The enhanced features $Y_{t}^{\left(i\right)}$ of this layer can be expressed as Equation (6):

(6) $$Y_{t}^{\left(i\right)}\to \left(Y_{t}^{i1},Y_{t}^{i2},\ldots ,Y_{t}^{iz}\right)=\left(p_{t}^{i1},q_{t}^{i1},p_{t}^{i2},q_{t}^{i2},\ldots ,p_{t}^{iz},q_{t}^{iz}\right),$$

where *z* is the number of forests in the layer. If there are *z* forests in this layer, the calculation equation of the enhanced feature number $N_{ef}$ of this layer is shown in Equation (7):

(7) $$N_{ef}=c^{*}z.$$

Among them, *c* represents the number of categories of the outcome variable. The enhanced features obtained at this layer are connected with the original features to form a new feature and transfer to next layer. Therefore, the feature set of the *t*-th borrower at the *i*-th layer of the cascade structure can be expressed as Equations (8)–(10):

The feature set of the first layer is the original feature set $X_{t}^{\left(1\right)}$:

(8) $$X_{t}^{\left(1\right)}=\left(X_{t1},X_{t2},\ldots ,X_{tn}\right).$$

The first layer feature set connects the enhanced features $Y_{t}^{\left(1\right)}$ to generate the second layer feature set:

(9) $$X_{t}^{\left(2\right)}=\left(X_{t}^{\left(1\right)},Y_{t}^{\left(1\right)}\right)=\left(X_{t1},X_{t2},\ldots ,X_{tn},p_{t}^{11},q_{t}^{11},p_{t}^{12},q_{t}^{12},\ldots ,p_{t}^{1z},q_{t}^{1z}\right).$$

The number of features accepted by the second layer is

(10) $$N_{X_{t}^{2}}=n+1*N_{ef}.$$

The feature set of the *i*-th layer is connected to the enhanced features $Y_{t}^{\left(i\right)}$ to generate the feature set of the *i* + 1th layer:

(11) $$X_{t}^{\left(i+1\right)}\to \left(X_{t}^{\left(i\right)},Y_{t}^{\left(i\right)}\right)=\left(X_{t1},\ldots ,X_{tn},p_{t}^{11},q_{t}^{11},\ldots ,p_{t}^{1,z},q_{t}^{1,z},\ldots ,p_{t}^{i,1},q_{t}^{i,1},\ldots ,p_{t}^{i,z}q_{t}^{i,z}\right).$$

The equation for calculating the number of features accepted by the *i*+1 layer is shown in Equation (12):

(12) $$N_{X_{t}^{\left(i+1\right)}}=n+i*N_{ef}.$$

To reduce the risk of overfitting, the class vector generated by each forest is generated by *k*-fold cross-validation. Each sample will be used as *k*-1 training data to generate *k*-1 class vectors and then we average them to generate the final class vector as the enhancement feature of the next stage in the cascade. In the expansion process, after each new layer is expanded, the performance of the entire cascade will be estimated on the verification set. If there is no significant performance improvement, the training process will terminate automatically. Therefore, the number of intermediate stages in the cascade is automatically determined. Contrary to most DNNs models with fixed complexity, gcForest can determine the complexity of its model appropriately by terminating training, which makes gcForest adaptive to different sizes of training data.

In the original cascade structure, to encourage diversity, different types of forests are included. Because of the serious problem of imbalance of credit default discrimination data and the advantage of heterogeneous ensemble model in handling imbalanced samples [33], this article improves the cascade structure in gcForest and combines Logic regression and XGBoost algorithm to enrich the original base classifier categories of the cascade layer. The parameters are optimized by the enumeration method to further strengthen the model’s ability to recognize minority samples and reduce type II error.

To prove the effectiveness of the constructed model, comparisons were made with five single classifiers, five ensemble classifiers, and convolutional neural networks (CNNs) in deep learning. Single classifiers include KNN, Bayes, Support Vector Machine (SVM), Artificial Neural Network (ANN), and Decision Tree (DT). Among the five ensemble classifiers, Bagging, RF, GBDT, XGBoost, and LightGBM are typical isomorphic ensemble methods. This study uses 10-fold cross-validation to verify the effectiveness of the model. To evaluate the classification algorithm, the following three indicators are used: (1) type II error; (2) AUC; (3) accuracy. Through these measures, the best classification algorithm is used to determine the credit score of the borrower of the financial institution. It is worth noting that this article focuses on the evaluation indicator of the type II error.

## 4. Experimental Setup

### 4.1. Experimental Data Set

This section uses three real credit data sets from UCI public database to conduct empirical research. Specifically, three credit data sets of Japanese, Australian, and German are used to evaluate the performance of the proposed two-stage hybrid model. The details of the three data sets are shown in Table 1.

### 4.2. Data Preprocessing

In reality, credit data inevitably has data missing. Before building the model, it is necessary to preprocess missing data to improve the prediction performance of the model. Besides, to avoid the magnitude difference between the data from affecting the classification results, the data set should be standardized before the model is constructed. In this study, the data preprocessing includes the following three steps. The first is the filling of missing values; the second is scoring processing for categorical variables according to the default situation of each category; the third is data standardization. After preprocessing the original data through these steps, new data is obtained.

The multistep data preprocessing process is as follows:

(1) Missing value filling. Based on the types of missing data in the original data set, we use the mode category to replace the missing values for categorical variables, and we use the mean to replace the missing values for numeric variables [34].
(2) Scoring with qualitative variables. Categorical variables are scored according to the default situation of each category: the relationship between each value of the categorical variable and the probability of default is calculated. In short, the higher probability of default, the lower the score.
(3) Standardize data to eliminate dimensional differences between variables. This article uses the z-score standardization method to standardize the data. The z-score standardization method [34] is shown in Equation (13):
  (13) $$x^{\prime }=\frac{x-\bar{x}}{s},$$
  where $x^{\prime }$ represents the processed value, *x* is the original value, $\bar{x}$ denotes the mean of the feature, *s* stipulates the standard deviation of the feature.

In the three public data sets used in this article, the Australian data set and the German data set are all complete data sets, while the Japanese data set has certain missing values, and the missing values need to be filled. For categorical variables, the missing values are filled in by the mode category, and the missing values of the numeric variables are filled in by the mean of the corresponding variable.

After the missing values are processed on the data, the category variables of each data set are scored according to the default situation of each category value. We use the EXCEL pivot table to calculate the relationship between the values of the categorical variables and the probability of default. The higher probability of default, the lower the score, and the lower probability of default, the higher the score. For numerical variables, the third step z-score standardization method is used to standardize the data to eliminate dimensional differences between variables.

After multistep data preprocessing, the data set is divided into a training set (I) and a test set (I) according to the ratio of 8:2 in the first stage. It means 80% of the data is used to train the model, and 20% of the data is used to verify the effectiveness of the model. In the second stage, we use 10-fold cross-validation to further improve the performance of each model. The enumeration method is used to optimize the parameters of gcForest, taking into account the type II error, AUC, and accuracy.

### 4.3. Evaluation Indicators

To evaluate the performance of the model, this section uses three evaluation indicators, namely the type II error, AUC, and accuracy, which are based on the confusion matrix shown in Table 2. In this study, we take the lowest type II error, the highest AUC, and accuracy as the first, second, and third principles separately to comprehensively evaluate the default feature set and default discrimination model. The type II error indicates the proportion of default borrowers who are misjudged as nondefault borrowers. In the credit loan situation, type II error will cause more losses to banks and other financial institutions, so they should pay more attention to type II error. AUC is a tool for binary classification analysis. The larger the value, the better the performance of the classifier. Besides, because it has better robustness than accuracy, when comparing the performance of machine learning algorithms, AUC is considered to be a more appropriate performance evaluation indicator than accuracy [35]. Accuracy represents the proportion of good and bad borrowers that are correctly classified and measures the classification ability of the model.

The confusion matrix is widely used to evaluate the performance of classification models. According to the true category and predicted category of the sample, the data sample can be divided into four categories, namely true positive (TP), false positive (FP), false negative (FN), and true negative (TN). Based on the confusion matrix, the calculation equations for the accuracy and the type II error are shown in Equations (14) and (15):

(14) $$\;\mathrm{Accuracy}\;=\frac{TP+TN}{TP+FN+FP+TN},$$

(15) $$\;\mathrm{Type}\;\mathrm{II}\;\mathrm{error}\;=\frac{FN}{TP+FN}.$$

## 5. Experimental Results and Analysis

### 5.1. Analysis of Feature Selection Results

The three real credit data sets of Japanese, Australian, and German in the UCI database and the constructed two-stage hybrid default discrimination model are used for empirical research. The various methods involved in the experiment are implemented using R 4.0.2 and Python 3.8.5. In this model, after data preprocessing in the first stage (Section 4.2), five feature selection algorithms are applied, and the results of feature selection are evaluated according to type II error, AUC, and accuracy of Logistic regression. Table 3, Table 4 and Table 5 show the regression coefficients and the feature selection results of the five feature selection methods on the three data sets.

The five feature selection methods in this article have good interpretability. For the above five feature selection methods, if a feature has a regression coefficient and the corresponding significance level *p*-value is less than 5%, the feature has a significant impact on the borrower’s default status; if the regression coefficient is positive, then the increase in the value of this feature corresponds to the increase in the possibility of default, and for the feature with negative regression coefficient, the increase in the feature value corresponds to the decrease in the possibility of default. Taking the Japanese data set as an example, Table 3 shows that for Full-variable Logistic regression method, the characteristics, A4, A6, A9, A11, A13, A14 and A15, have a significant impact on the default status of the borrower. Among them, the regression coefficients of A9 and A14 are positive, and their increase corresponds to an increase in the probability of default; the regression coefficients of A4, A6, A11, A13 and A15 are negative, therefore their increase corresponds to a decrease in the probability of default. For Stepwise regression based on AIC criterion, the retained features are exactly the same as those retained by the Full-variable Logistic regression, and the coefficients are not much different. In Stepwise regression based on BIC criterion, the predictive variables that have a significant impact on the borrower’s default behavior are A6, A9, A11, A15. The Stepwise regression based on BIC criterion eliminates the three characteristics, A4, A13, and A14, based on the Full-variable Logistic regression. Elastic Net Logistic regression retains a total of 11 features, including A4, A5, A6, A7, A8, A9, A10, A11, A13, A14 and A15. Lasso-logistic regression retains a total of seven features and eliminates A7, A8, A13, A14 based on the Elastic Net Logistic regression. Compared with Elastic Net Logistic regression, the Lasso-logistic regression is more concise. In summary, on the Japanese data set, four feature selection methods all believe that the five features A6, A9, A11, and A15 have a significant impact on the default status, while the features, A1, A2, A3 and A12, have no significant impact on the default status. This shows that feature selection for the Japanese data set is necessary, which can help us select features that have significant default identification capabilities for borrowers, and can eliminate some less useful features and improve the interpretability of the feature system. Analysis of the Australian data set and the German data set in Table 4 and Table 5 can conclude similar conclusions to the Japanese data set.

Table 6 shows the type II error, AUC, and accuracy of five feature selection methods on the test set of three data sets. Obviously, the lower the type II error, the greater the AUC and accuracy, and the better the effect of the feature system. According to the constructed five groups of default discrimination feature sets, the type II error is the first principle, and the highest AUC and accuracy are the second and third principles to select a set of optimal default discrimination feature sets.

From the comparison of the results of five feature selection methods in the Japanese data set in Table 6, the Lasso-logistic regression performs best on the Japanese data set. The type II error measures the probability of predicting a defaulting borrower as a nondefaulting borrower. The lower the value, the better the model. The type II error of the Lasso-logistic model is 0.0909, the AUC is 0.9619, and the accuracy is 0.9203, which are better than other feature selection methods. Therefore, on the Japanese data set, the feature system constructed by Lasso-logistic regression is considered to be the feature set with the best default identification ability. On the Australian data set, the Lasso-logistic regression has a better predictive effect. From the perspective of the type II error, Full-variable Logistic regression, Lasso-logistic regression, and Elastic Net Logistic regression are all 0.1781, which is lower than the two stepwise regression models. For the AUC, the Lasso-logistic regression is 0.9444, which is slightly better than other methods. It is 0.0015 higher than the Elastic Net Logistic regression and 0.0114 higher than the Full-variable Logistic regression. The AUC of the Lasso-logistic regression is higher than that of the Full-variable Logistic regression, and the accuracy is lower than that of the Full-variable Logistic regression. Therefore, on the Australian data set, the feature system constructed by Lasso-logistic regression is considered to be the credit scoring feature system with the best ability to identify defaulters. Based on the first principle is the lowest type II error, and the second and third principles are the highest AUC and accuracy, the feature selection results on the German data set show that the prediction performance of the Stepwise regression based on AIC criterion is better. The Full-variable Logistic regression and the Stepwise regression based on AIC criterion have the lowest type II error. On the evaluation indicator of AUC, the Stepwise regression based on AIC criterion performs better. Therefore, the feature system constructed by the Stepwise regression based on AIC criterion is selected as the optimal default discrimination feature set of the German data set.

### 5.2. Analysis on the Results of Ddefault Discrimination

We used three real credit data sets of Japanese, Australian, and German in the UCI database, and combined with the optimal feature set with good interpretability selected in the first stage, and then constructed a default discrimination model using gcForest. The enumeration method was used to simultaneously take into account the three goals, the first principle is the lowest type II error, and the second and third principles are the highest AUC and accuracy, to optimize the parameters of gcForest. This part of the experiment uses Python 3.8.5 for demonstration. The computer processor is i7-10700, and the memory is 48 G.

Table 7 shows the running results and computational times of the gcForest and other 11 common classifiers on the data sets of Japanese, Australian, and German. To evaluate the model comprehensively, three evaluation indicators of type II error, AUC, and accuracy are used, and the top three classifiers on each evaluation indicator are highlighted in bold. On the Japanese data set, the performance of the gcForest is better than the current popular single classifiers such as ANN, and the common ensemble classifiers such as LightGBM, and CNNs in type II error, AUC, and accuracy. Type II error of gcForest is 0.0500, which is 4.09% lower than that of the first stage. Type II error is significantly reduced through effective identification of defaulting borrowers, which can further help banks and other financial institutions reduce the possible default losses of borrowers. On the Australian data set, comparing with the popular ANN, RF, XGBoost, and CNNs, gcForest has the lowest type II error, which is 0.0553; compared with type II error of 0.1781 in the first stage, gcForest has decreased by 12.28%. In terms of AUC and accuracy, the performance of gcForest is slightly inferior to other ensemble algorithms such as GBDT, but it still maintains a high performance. On the German data set, gcForest’s performance is the best in the three evaluation indicators of type II error, AUC, and accuracy. Type II error in the second stage is 0.2942, which is a 16.03% drop compared to 0.4545 in the first stage, and the AUC and accuracy are increased by 4.80% and 2.20%, respectively. From this data, gcForest further improves the ability to discriminate defaults of borrowers based on the first-stage model. In summary, on the three data sets in the UCI database, the overall discriminative performance of the gcForest can maintain the best or the second-best. Compared with other models, it has better robustness and can be better adapted to different data sets. From the results in Table 7, CNNs and ensemble algorithms such as GBDT have better performance, but from the perspective of robustness, they are slightly inferior to the gcForest.

From the last column in Table 7, CNNs have the longest computational times, followed by gcForest and ANN. The average computational time of the three algorithms on the data sets of Japanese, Australian, and German is 156.43 h, 85.60 h, and 36.17 h respectively. The shortest computational time is NB, followed by KNN and DT. The average computational time of the three algorithms is 0.01 h, 0.02 h, and 0.04 h respectively. The shorter computational time is SVM, RF, and Bagging. The average computational time of the three algorithms is 0.98 h, 2.64 h, and 3.06 h respectively. Although gcForest has a longer computational time, the overall performance of gcForest is the best. In particular, financial institutions such as banks pay more attention to type II error, and they can use multiple servers in parallel to shorten computational time. NB, KNN, and DT require just minutes and obtain good performance. Low computational time can be a plus when data analysts need the result soon.

**Figure 2:**
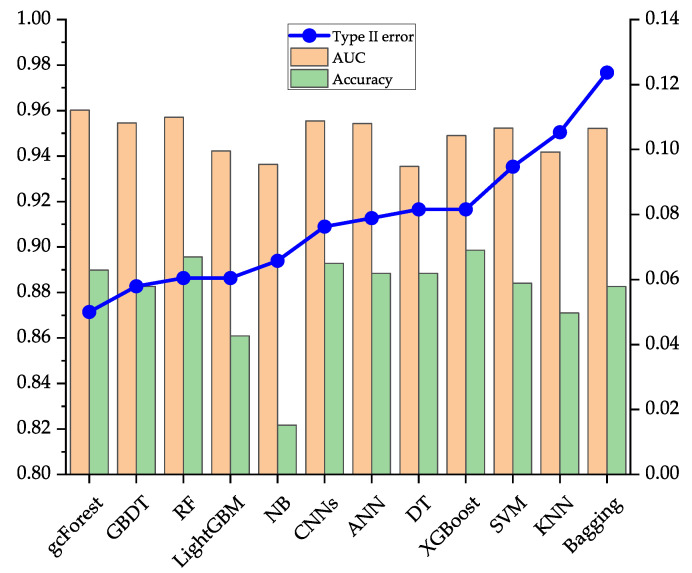
Evaluation results of 12 classification models on the Japanese data set.

**Figure 3:**
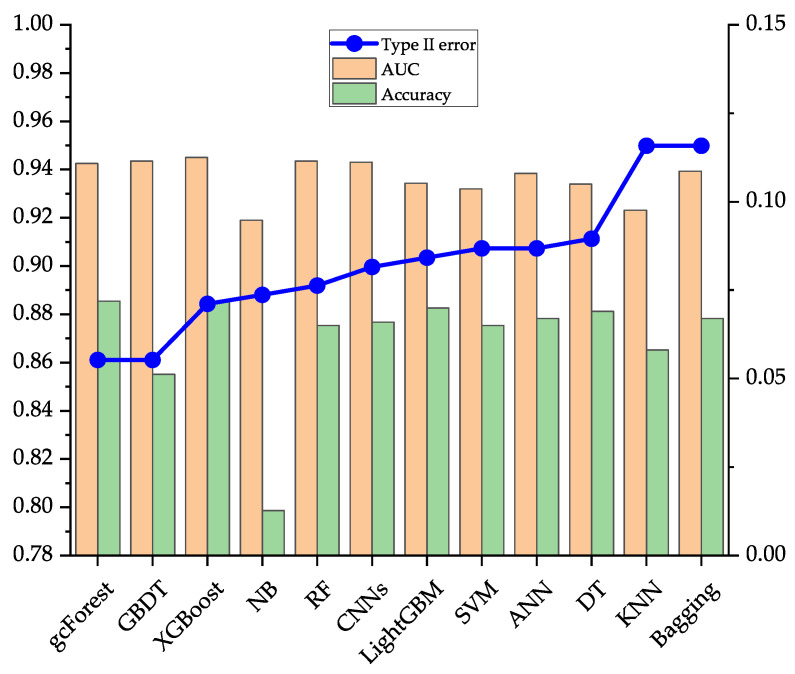
Evaluation results of 12 classification models on the Australian data set.

**Figure 4:**
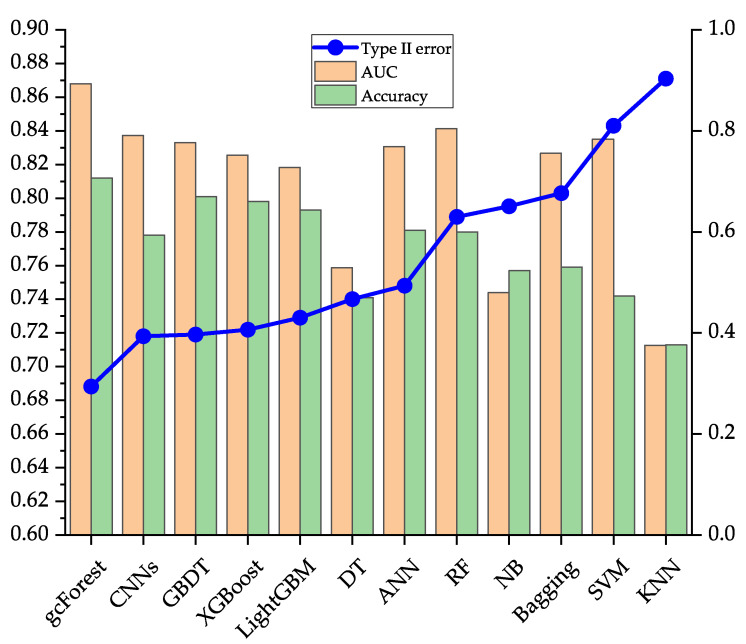
Evaluation results of 12 classification models on the German data set.

Figure 2, Figure 3 and Figure 4 compare the performance of the 12 classification models on the three data sets on type II error, AUC, and accuracy more intuitively. In this article, we focus on the evaluation indicator of the type II error, so Figure 2, Figure 3 and Figure 4 are obtained after sorting the classification models according to type II error from low to high.

Figure 2 shows the performance of 11 comparative classification models and gcForest on the Japanese data set. The smaller the type II error, the higher the performance of the classification model. However, AUC and accuracy are opposite to type II error. These two indicators show the same trend. When the value of the two indicators of a classification model is larger, the overall classification loss will be smaller. It can be seen from Figure 2 that, compared with other classifiers, gcForest has the best performance in the two evaluation indicators of type II error and AUC but has the slightly inferior performance in accuracy.

Figure 3 shows the performance of five single classifiers, five ensemble classifiers, a deep learning model, and a gcForest model on the Australian data set. From the perspective of type II error and accuracy, gcForest has better performance than other models. However, indicator of AUC in gcForest is slightly inferior to ensemble models such as GBDT.

It can be seen from Figure 4 that on the German data set, gcForest has the lowest type II error and the highest AUC and accuracy, indicating its excellent overall discrimination performance.

All in all, gcForest not only has the best performance in accurately identifying default borrowers but also maintains the best or second-best overall discrimination performance in most cases. Compared with other models, it maintains a higher discrimination ability and ensures the robustness. Therefore, gcForest is more suitable as an effective tool for banks and other financial institutions to distinguish potential defaulting borrowers.

### 5.3. Comparison with Other Studies

Section 5.2 proves the discriminative accuracy and robustness of the constructed two-stage hybrid model through empirical results on three real credit data sets. To further verify the applicability and effectiveness of the model in the credit field, this section compares the research results of other researchers on the same credit loan data set. The specific results are shown in Table 8. The comparison results show that compared with other models, the prediction performance of this model on different data sets can achieve better results, and the prediction results on different data sets are more robust.

## 6. Conclusions

Establishing a borrower’s default discrimination model is an important task for banks and other financial institutions to make loan decisions. Therefore, the discriminative performance, interpretability, and robustness of the default discrimination model are crucial to the profitability of banks and other financial institutions. In this study, we combine a statistical model with good interpretability and an artificial intelligence model with better predictive performance to construct a hybrid default discrimination model.

Firstly, we choose five traditional statistical methods with good interpretability to construct a feature selection model, including (1) Full-variable Logistic regression; (2) Stepwise regression based on AIC criterion; (3) Stepwise regression based on BIC criterion; (4) Lasso-logistic regression; (5) Elastic Net Logistic regression. The feature selection is performed separately, constructing five groups of different default discrimination feature sets. This paper takes type II error as the first principle, the highest AUC and accuracy as the second and third principles respectively, and evaluates five groups of feature systems, so as to select a set of optimal default discrimination features that are most suitable for the data set.

Secondly, based on the optimal default discrimination feature set constructed in the first stage, we combine with gcForest to construct a personal credit default discrimination model. Existing studies show that gcForest has excellent predictive performance in many fields such as medicine. GcForest uses cascade processing to effectively identify and process high-dimensional feature information. At the same time, gcForest has fewer hyperparameters and strong robustness. Taking into account the imbalance of data in the credit default discrimination data and the advantages of heterogeneous ensemble models in handling imbalanced samples, this articel modifies the cascade structure in gcForest, and combines Logistic regression and XGBoost algorithm to enrich the original base classifier categories in cascade layer and further improve the prediction performance and robustness of the model. The enumeration method is used to adjust the model parameters at the same time, taking into account type II error, AUC, and accuracy, to further strengthen the entire forest’s ability to recognize minority samples and reduce type II error.

Finally, we use three real open credit data sets in the UCI database, including Australian, Japanese, and German, to verify the performance of the hybrid model constructed in this article. From three aspects of type II error, AUC, and accuracy, gcforest is compared with single classifier, ensemble classifier, and deep learning model. To further prove the effectiveness of the proposed model, gcForest is compared with other models of existing research on the same data set. The results show that the hybrid default discrimination model has better interpretability, discrimination accuracy, and robustness than other single classifiers, ensemble classifiers, and deep learning model.

In addition, this study has some limitations. Firstly, in this study, we adopt a preprocessing step to fill in missing values and standardize data. Therefore, the first research direction is to compare the nonprocessing of missing values and standardization, and we will further discuss these steps and estimate how much they influence the classification performance. Secondly, in the first stage, the machine learning algorithm is not used to select feature. Therefore, the second research direction is to use machine learning algorithms (i.e., XGBoost), which is guided by the lowest type II error of default prediction, retain the features with higher importance, and reverse the optimal feature set. Thirdly, in this research, we only try four kinds of base classifiers in gcForest. Therefore, in further exploration, more base classifiers (i.e., CNNs algorithm) should be used in gcForest, which may lead to better performance.

## Figures and Tables

**Table 1:** Description of the three data sets used in the study.

Data Set	Samples	Good	Bad	Features	Category Features	Numerical Features
Japanese	690	307	383	15	11	4
Australian	690	307	383	14	6	8
German	1000	700	300	20	13	7

**Table 2:** Confusion matrix for credit scoring.

		Predicted	
		Positive (Non-Risk)	Negative (Risk)
Real	Positive (Non-Risk)	True Positive (TP)	False Negative (FN)
	Negative (Risk)	False Positive (FP)	True Negative (TN)

**Table 3:** The regression coefficients and feature selection results of the 5 feature selection methods on the Japanese data set.

Variable	Full-LR		AIC		BIC		Lasso-LR		EN-LR	
	Coef	Whether Keep	Coef	Whether Keep	Coef	Whether Keep	Coef	Whether Keep	Coef	Whether Keep
A1	0	–	0	–	0	–	0	–	0	–
A2	0	–	0	–	0	–	0	–	0	—
A3	0	–	0	–	0	–	0	–	0	–
A4	−3.91	keep	−3.86	keep	0	–	−0.48	keep	−0.56	keep
A5	0	–	0	–	0	–	−3.47	keep	−0.54	keep
A6	−2.53	keep	−2.50	keep	−2.63	keep	−1.31	keep	−1.35	keep
A7	0	–	0	–	0	–	0	–	−0.03	keep
A8	0	–	0	–	0	–	0	–	−0.09	keep
A9	13.17	keep	13.15	keep	12.56	keep	10.96	keep	9.14	keep
A10	0	–	0	–	0	–	−2.41	keep	−2.58	keep
A11	−0.95	keep	−0.95	keep	−1.05	keep	−0.20	keep	−0.25	keep
A12	0	–	0	–	0	–	0	–	0	–
A13	−6.51	keep	−6.51	keep	0	–	0	–	−0.82	keep
A14	0.37	keep	0.39	keep	0	–	0	–	0.01	keep
A15	−3.00	keep	−2.97	keep	−2.73	keep	−0.11	keep	−0.13	keep

**Table 4:** The regression coefficients and feature selection results of the 5 feature selection methods on the Australian data set.

Variable	Full-LR		AIC		BIC		Lasso-LR		EN-LR	
	Coef	Whether Keep	Coef	Whether Keep	Coef	Whether Keep	Coef	Whether Keep	Coef	Whether Keep
A1	0	–	0	–	0	–	0	–	0	–
A2	0	–	0	–	0	–	0	–	0	–
A3	0	–	0	–	0	–	0	–	0	–
A4	−2.34	keep	−2.29	keep	−2.23	keep	−0.48	keep	−0.64	keep
A5	−3.94	keep	−3.77	keep	−3.92	keep	−2.13	keep	−2.10	keep
A6	0	–	0	–	0	–	0	–	−0.13	keep
A7	0	–	−0.36	keep	0	–	−0.02	keep	−0.10	keep
A8	−4.82	keep	−4.77	keep	−4.77	keep	−3.98	keep	−3.42	keep
A9	0	–	0	–	0	–	−1.07	keep	−1.18	keep
A10	−0.69	keep	−0.79	keep	−0.82	keep	−0.28	keep	−0.29	keep
A11	4.18	keep	4.58	keep	0	–	0	–	0	–
A12	0	–	0	–	0	–	0	–	0	–
A13	0	–	0	–	0	–	0.01	keep	0.06	keep
A14	−2.26	keep	−2.25	keep	−2.39	keep	−0.16	keep	−0.18	keep

**Table 5:** The regression coefficients and feature selection results of 5 feature selection methods on the German data set.

NO.	Variable	Full-LR		AIC		BIC		Lasso-LR		EN-LR	
		Coef	Whether Keep	Coef	Whether Keep	Coef	Whether Keep	Coef	Whether Keep	Coef	Whether Keep
1	Duration in month	0.23	keep	0.24	keep	0.45	keep	0.29	keep	0.24	keep
2	Credit amount	0.33	keep	0.34	keep	0	–	0.02	keep	0.08	keep
3	Installment rate in percentage of disposable income	0.37	Keep	0.37	keep	0	–	0.09	keep	0.11	keep
4	Present residence since	0	–	0	–	0	–	0	–	0	–
5	Age in years	−0.21	keep	−0.16	keep	0	–	−0.03	keep	−0.06	keep
6	Number of existing credits at this bank	0	–	0	–	0	–	0	–	0	–
7	Number of people being liable to provide maintenance for	0	–	0	–	0	–	0	–	0	–
8	Status of existing checking account	−3.86	keep	−3.91	keep	−3.97	keep	−3.69	keep	−3.16	keep
9	Credit history	−4.00	keep	−3.82	keep	−4.18	keep	−2.47	keep	−2.28	keep
10	Purpose	−5.65	keep	−5.74	keep	−5.27	keep	−3.02	keep	−2.81	keep
11	Savings account bonds	−4.03	keep	−4.01	keep	−3.88	keep	−2.36	keep	−2.33	keep
12	Present employment since	−3.52	keep	−3.10	keep	−3.67	keep	−1.79	keep	−1.83	keep
13	Personal status and sex	−5.92	keep	−5.44	keep	0	–	−1.88	keep	−2.17	keep
14	Other debtors guarantors	−4.48	keep	−4.59	keep	0	–	−1.82	keep	−1.95	keep
15	Property	0	–	0	–	0	–	−0.78	keep	−0.98	keep
16	Other installment plans	−3.63	keep	−3.85	keep	0	–	−1.24	keep	−1.61	keep
17	Housing	0	–	−2.85	keep	0	–	−0.65	keep	−0.97	keep
18	Job	0	–	0	–	0	–	0	–	0	–
19	Telephone	−15.9	keep	−14.7	keep	0	–	0	–	−0.88	keep
20	foreign worker	−7.75	keep	−7.97	keep	0	–	−1.73	keep	−2.09	keep

**Table 6:** Comparison of 5 feature selection methods on 3 data sets.

Data Set	Feature Selection Method	Evaluation Indicator		
		Type II Error	AUC	Accuracy
Japanese	Full-LR	0.1169	0.9520	0.8841
	AIC	0.1039	0.9530	0.8986
	BIC	0.1299	0.9550	0.8913
	**Lasso-LR**	**0.0909**	**0.9619**	**0.9203**
	EN-LR	0.0909	0.9615	0.9130
Australian	Full-LR	0.1781	0.9330	0.8768
	AIC	0.1918	0.9280	0.8696
	BIC	0.1918	0.9350	0.8623
	**Lasso-LR**	**0.1781**	**0.9444**	**0.8696**
	EN-LR	0.1781	0.9429	0.8696
German	Full-LR	0.4545	0.8140	0.7900
	**AIC**	**0.4545**	**0.8200**	**0.7900**
	BIC	0.4727	0.8040	0.8000
	Lasso-LR	0.4909	0.8350	0.8050
	EN-LR	0.4909	0.8342	0.8000

**Table 7:** Evaluation results of 12 classification models on UCI data set.

Data Set	Classifier	Evaluation Inditcator			Total Time (h)
		Type II error	AUC	Accuracy	
Japanese	KNN	0.1053	0.9418	0.8710	0.02
	NB	0.0658	0.9363	0.8217	0.01
	SVM	0.0947	0.9523	0.8841	0.87
	ANN	0.0789	0.9543	0.8884	32.10
	DT	0.0816	0.9354	0.8884	0.04
	Bagging	0.1237	0.9522	0.8826	2.68
	RF	0.0605	**0.9570**	**0.8957**	2.37
	GBDT	**0.0579**	0.9545	0.8826	20.07
	XGBoost	0.0816	0.9490	**0.8986**	30.58
	LightGBM	**0.0605**	0.9422	0.8609	10.69
	CNNs	0.0763	**0.9555**	**0.8928**	135.14
	gcForest	**0.0500**	**0.9602**	0.8899	76.65
Australian	KNN	0.1158	0.9231	0.8652	0.02
	NB	0.0737	0.9191	0.7986	0.01
	SVM	0.0868	0.9320	0.8754	0.85
	ANN	0.0868	0.9385	0.8783	34.67
	DT	0.0895	0.9340	0.8812	0.04
	Bagging	0.1158	0.9393	0.8783	3.05
	RF	0.0763	**0.9435**	0.8754	2.66
	GBDT	**0.0553**	**0.9435**	0.8551	24.58
	XGBoost	**0.0711**	**0.9450**	**0.8855**	34.03
	LightGBM	0.0842	0.9343	**0.8826**	11.37
	CNNs	0.0816	0.9431	0.8768	145.76
	gcForest	**0.0553**	0.9425	**0.8855**	81.12
German	KNN	0.9033	0.7126	0.7130	0.03
	NB	0.6504	0.7439	0.7570	0.01
	SVM	0.8100	0.8350	0.7420	1.22
	ANN	0.4933	0.8306	0.7810	41.73
	DT	0.4667	0.7588	0.7410	0.05
	Bagging	0.6767	0.8268	0.7590	3.46
	RF	0.6300	**0.8413**	0.7800	2.90
	GBDT	**0.3967**	0.8330	**0.8010**	32.16
	XGBoost	0.4067	0.8255	**0.7980**	40.30
	LightGBM	0.4300	0.8183	0.7930	15.46
	CNNs	**0.3933**	**0.8373**	0.7780	188.39
	gcForest	**0.2942**	**0.8680**	**0.8120**	99.03

**Table 8:** Performance comparisons with other default discrimination models.

Model	Evaluation Indicator	Japanese	Australian	German
Our proposed model	Type II error	0.0500	0.0553	0.2942
	AUC	0.9602	0.9425	0.8680
	Accuracy	0.8899	0.8855	0.8120
Zhang et al.’s work (2020) [36]	Type II error	–	–	–
	AUC	0.9696	0.9666	0.8312
	Accuracy	0.9316	0.9236	0.7950
Papouskova et al.’s work (2019) [37]	Type II error	–	–	–
	AUC	–	0.9280	0.7948
	Accuracy	–	0.8828	0.7866
Guo et al.’s work (2019) [38]	Type II error	–	–	–
	AUC	0.9420	0.9400	0.8060
	Accuracy	0.8700	0.8740	0.7830
Zhang et al.’ work (2019) [19]	Type II error	–	–	–
	AUC	0.9387	0.9370	0.8029
	Accuracy	0.8720	0.8754	0.7682

## Data Availability

We provide raw data, please click http://archive.ics.uci.edu/ml/datasets/Credit+Approval (accessed on 5 October 2020) for Japanese data set, http://archive.ics.uci.edu/ml/datasets/Statlog+%28Australian+Credit+Approval%29 (accessed on 5 October 2020) for Australian data set, and http://archive.ics.uci.edu/ml/datasets/Statlog+%28German+Credit+Data%29 (accessed on 5 October 2020) for German data set.

## Abbreviations

The following abbreviations are used in this manuscript:

ACC	accuracy
AIC	Stepwise regression based on AIC criterion
ANN	artificial neural network
AUC	the area under the Receiver Operating Characteristic (ROC) curve
Bagging	Bootstrap aggregating
BIC	Stepwise regression based on BIC criterion
CNNs	the convolutional neural networks
Coef	coefficient
DNNs	the deep neural networks
DT	decision tree
EN-LR	Elastic Net Logistic regression
ET	Extra-Trees
Full-LR	Full-variable Logistic regression
FS	feature selection
GBDT	Gradient Boosting Decision Tree
gcForest	the multi-Grained Cascade Forest
KNN	k-Nearest Neighbor
Lasso-LR	Lasso-logistic regression
LDA	Linear Discriminant Analysis
LR	Logistic regression
NB	Naive Bayes
RF	Random forest
SVM	support vector machines
XGBoost	eXtreme Gradient Boosting
